# Environmental impact of infant feeding type, accessories used and maternal dietary habits: The GREEN MOTHER-I project, a cross-sectional study protocol

**DOI:** 10.1186/s12937-024-01000-9

**Published:** 2024-08-21

**Authors:** Rosa Maria Cabedo-Ferreiro, Liudmila Liutsko, Judit Cos-Busquets, Rosa García-Sierra, Margalida Colldeforns-Vidal, Azahara Reyes-Lacalle, Mª Mercedes Vicente-Hernández, Miriam Gómez-Masvidal, Laura Montero-Pons, Gemma Cazorla-Ortiz, Pere Torán-Monserrat, Concepció Violán, Gemma Falguera-Puig, The GREEN MOTHER Group, Glòria Seguranyes-Guillot, Josep Mª Manresa-Dominguez, Anna Perez-Llusà, Antonia Arias-Perianez, Roser Gol-Gómez, Mª Dolores Alcaraz-Sanz, Núria Nebot-Rodrigo, Salut Puig-Calsina, Lucia Alcaraz-Vidal, Núria Sitjà-Begué, Ana M. Barluenga Perez-Cossio, Miriam Garcia-Sanchez, Esther Cerro-Hernandez, Cristina Morote-Muñoz, Paula Amoros-Ferrer, Raquel Martinez-Mondejar, Natalia Dueñas-Herrero, Marta Xivillé-Sole, Marina Raja-Carcaña, Núria Risques-Fernandez, Anna Vila-Corominas, Assumpta Prats-Oliveras, Susanna Sancho-Esteban, Mercedes Giselle Vigil-Mamani, Carmen Barrionuevo-Ramírez, Antònia Arias-Perianez, Marta Casquete-Perez, Nuria Buitrago-Torrijos, Gladis Margarita Maldonado-Aubian, Maria Camacho-Donézar, Inés María García-Martín, Sara Nieto-Tirado, Cristina Olivas-Menendez, Maria Inmaculada Rodriguez-Alvarez, Lucia Burgos-Cubero, Júlia Gonzalo-Ortega, David Porras-Paradas, Tamara Salceda-Varea, Roser Samsó-Julià, Rita Suñé-Socias, Mª Carmen Vidal-Testal, Carolina Alós-Rodriguez, Montse Garrido-Dominguez, Glòria Miralpeix-Pomar, Irene Fernandez-Varela, Ariadna Torres-Franco, Gemma Prieto-Sanchez, Mercedes Guerrero-Martinez, Margarita Mendoza-Ariza, Meritxell Fors-Andreu, Carolina Expósito-Moreno, Silvia Martinez-Rubiño, Sara Perez-Navarro, Rocío Rodríguez-López, Cristina Garcia-Gonzalez, Laura Cristóbal-Balbás, Aleida Ribas-Tristany, Raquel Antón de Silva, Elena Imbernon-Bustamante, Anna Estruch-Riu, Ainhoa Borras-Reverter, Alba Llobera-Sanz, Paloma Amado-Barroso, Soraya Vera-Pérez, Miriam Gómez-Masvidal, Marina Martinez-Diaz, Saray Gonzalez-Perez, Montserrat Pujol-Abajo, Mireia Monllau-Ros, Mercè Sesa-Nogueras, Rosa Tamaral-Cepas, Yolanda Tortola-Brocal, Marta Guillen-Vila, Laura Muñoz-Tamajon, Alba Garcia-Muñoz, Mònica Martinez-Terron, Eduard Lobera Gutierrez de Pando, Lorena Segovia-Navarro, Eva Bueno-López, Antonio López-Ollero, Concepción de la Fuente Guirado, Laura Tarrats-Velasco, Montserrat Garrido-Domingo, Susana Calle del Fresno, Meritxell Casajoana-Guerrero, Míriam Segura-Soler, Meritxell Gomez-Maldonado, Jose Cano-Blasco

**Affiliations:** 1 Atenció a La Salut Sexual I Reproductiva (ASSIR) Granollers, Institut Català de La Salut (ICS), Barcelona, Spain; 2 Research Group On Sexual and Reproductive Healthcare (GRASSIR), 2021-SGR-793), Barcelona, Spain; 3 Atenció a La Salut Sexual I Reproductiva (ASSIR) Sabadell, ICS, Sabadell, Spain; 4 Institut Universitari d’Investigació en Atenció Primària Jordi Gol (IDIAP Jordi Gol), Unitat de Suport a La Recerca Metropolitana Nord, Mataró, Spain; 5 Multidisciplinary Research Group in Health and Society (GREMSAS), 2021-SGR-0148) Barcelona, Spain; 6 Atenció a La Salut Sexual I Reproductiva (ASSIR) Badalona - Sant Adrià, ICS, Sant Adrià del Besós, Spain; 7 Atenció a La Salut Sexual I Reproductiva (ASSIR) Mataró, ICS, Mataró, Spain; 8 Atenció a La Salut Sexual I Reproductiva (ASSIR) Santa Coloma de Gramanet, Primary Care Management Metropolitana Nord, Catalan Institute of Health, Santa Coloma de Gramanet, Barcelona, Spain; 9 Doctoral Program in Nursing and Health, Faculty of Medicine and Health Sciences, School of Nursing, Universitat de Barcelona, L’Hospitalet de Llobregat, Barcelona, Spain; 10 Institut d’Investigació Germans Trias I Pujol (IGTP), Badalona, Spain; 11 Department of Medicine, Faculty of Medicine, Universitat de Girona, Girona, Spain; 12 Universitat Autònoma de Barcelona, Cerdanyola del Vallès, Spain; 13 Grup de Recerca en Impacte de Les Malalties Cròniques I Les Seves Trajectòries (GRIMTra), Institut Universitari D’Investigació en Atenció Primària Jordi Gol (IDIAPJGol), Barcelona, 2021 SGR 01537 Spain; 14 El Grupo de Investigación en Servicios Sanitarios en Atención Primaria (GrenSSAP), Institut Universitari D’Investigació en Atenció Primària Jordi Gol (IDIAPJGol), RICAPPS, Barcelona, RD21/0016/0029), Spain

**Keywords:** Public health, Postpartum women, Nutrition and dietetics, Climate change, Environmental impact, Environmental health, Breastfeeding, Infant feeding

## Abstract

**Introduction:**

Breastfeeding (BF) is the healthiest form of nutrition for babies and is recommended exclusively (EBF) for at least the first six months of life. The carbon footprint of formula feeding (FF) has been studied, but that of BF is unknown.

**Aim:**

To identify the environmental impact of three types of infant feeding taking into account the accessories needed and the diet of postpartum women in the baby’s first month of life.

**Methods:**

This is a multicentre, cross-sectional study conducted in the Barcelona North Metropolitan Area (Catalonia, Spain). The participating sites are primary care settings that will recruit 408 postpartum women (4–6 weeks) as per inclusion/exclusion criteria. The data will be collected through a GREEN MOTHER Survey that includes 4 dimensions: 1) socio-demographic and clinical data; 2) data on the newborn and accessories used in infant feeding; 3) general data on the mother's diet (food consumption habits), and 4) recording of 24 h of the mother’s diet. The data analysis will be performed to check the prevalence of infant feeding types at birth and month 1, as well as a comparative analysis of three types of infant feeding on environmental impact (climate change; water consumption, and scarcity).

Ethics.

This project was approved by the Research Ethics Committee of the Jordi Gol i Gurina University Institute Foundation for Primary Health Care Research (IDIAP) under code 22/101-P dated 22/02/2023.

**Discussion:**

A second phase of the GREEN MOTHER study is planned, which will consist of an educational intervention to promote breastfeeding, nutrition and sustainability. This intervention will be based on the results obtained in Phase I. We expect that the project results – through the publication and dissemination of scientific papers and reports among relevant stakeholders (association of community midwives, healthcare and primary care attention professionals and the public) – will increase public awareness of breastfeeding and its impact on sustainability.

**Trial registration:**

Both phases of the GREEN MOTHER study protocol were registered in ClinicalTrials.gov, NCT05729581.

**Supplementary Information:**

The online version contains supplementary material available at 10.1186/s12937-024-01000-9.

## Background

Breastfeeding (BF) is an eco-friendly, sustainable, and non-polluting form of feeding compared to formula feeding (FF) (for definitions of infant feeding types, see Annex I). It results in less waste production, minimal greenhouse gas (GHG) emissions, a smaller carbon footprint (see definitions of the environmental impacts referred to in this article in Annex I), and smaller water footprint (WF) [1, 2]. According to the Convention on the Rights of the Child [3], all infants and children have the right to the enjoyment of the highest attainable standard of health. The World Health Organization (WHO) recommends that BF be exclusively used for the first six months of life and that it be continued up to two years of age or for as long as the mother and child wish. BF offers multiple physical and mental health benefits for both mother and child and is environmentally friendly [2–4].

Some of the short-term benefits to children’s health include reduction of neonatal mortality, protection against gastrointestinal and respiratory infections, dental malocclusion and atopy [5–7]. In the long term, children and teenagers who were breastfed are less likely to be overweight or obese, are more likely to have healthier cognitive development and score better on intelligence tests, are associated with a higher income as adults, and are less likely to suffer diabetes or cardiovascular disease throughout their lives [4, 6]. Longer exclusive breastfeeding (EBF) also contributes to the health and well-being of mothers [4, 5]. It reduces the risk of ovarian and breast cancer, helps space out pregnancies, and decreases the risk of type 2 diabetes [4–6]. Improved child development and reduced healthcare costs thanks to BF result in financial savings for families, the nation, and society in general [2].

Under the Paris Agreement, the European Commission has set ambitious goals to reduce the continent’s carbon footprint, including a 55% reduction in GHG emissions by 2030 and climate neutrality by 2050 [8].

The manufacture and distribution of industrial infant formula is harmful to the environment, generates polluting waste, and requires energy use to manufacture the formula and packaging materials, and to transport the products, and water use to prepare bottles on a daily basis. Most infant formulas are based on cow's milk. Dairy farms represent the main source of anthropogenic methane emissions. In Europe, livestock farming represents 12–17% (630–863 Mt CO_2_-eq) of total GHG emissions [8].

For an accurate calculation of the environmental impact of breastfeeding, the infant feeding accessories (breast milk pump; milk storage containers or bags, bottles, bottle warmer, and bottle sterilizer, for example) and a theoretical increase of 500 kcal in the diet of the mother must be taken into account [1, 9–12].

There is data on the carbon footprint and water footprint of FF, but there is none on the environmental impact of breastfeeding. The average carbon footprint of FF was estimated to be 9.2, 7, 8.4, and 11 kg CO_2_-eq per kg of formula in New Zealand, USA, Brazil, and France, respectively. The potential carbon savings of BF compared to FF for the UK, China, Brazil and Vietnam is 40–55% [1].

The additional volume of water that a mother consumes while breastfeeding is minor compared to the significant quantities needed for manufacturing formula milk [12]. Research conducted by Pope et al. in the United States demonstrated that producing 1 kg of commercial formula requires 6.6 kg of raw milk, which entails a water footprint (WF) comprising 626 L of blue water for manufacturing, reconstitution, and sterilization, 6,280 L of green water for cow feed, and 524 L of grey water [13]. In Switzerland, Rollins and colleagues estimated that the average WF for whole cow's milk is around 940 L of water per kg of raw milk. When considering that 1 kg of whole milk produces about 200 g of milk powder, the water consumption (WC) amounts to 4,700 L of water per 1 kg of milk powder [14, 15].

Globally, water scarcity presents a significant challenge in fulfilling the need for freshwater for drinking and agricultural purposes [16]. In Spain, food consumption is associated with a water wastage of 3,302 L per person per day. The most substantial environmental impact from water use is attributed to the production of meat, fish, and animal fats (26%), as well as dairy products (21%) [17]. The water footprint of food waste was estimated to be 2,095 hm^3^, equivalent to 131 L per person per day [17]. Given the heightened water stress, this study also assesses the water stress implications of the life cycle of formula production, infant feeding accessories, and maternal dietary practices. There is currently no rigorous data on the GHG savings that Europe could achieve by taking into account the dietary habits of postpartum mothers if BF was their top choice rather than FF. To meticulously compare the contribution of EBF and FF to global warming in terms of their carbon footprint, the various dietary patterns of postpartum women and the accessories required for breastfeeding must be considered. Although there is no consensus on the need to supplement the diet of lactating mothers, it is recommended that they increase their daily calorie intake by 500 kcal to cover the nutritional and energy requirements of feeding their baby [18, 19]. Additionally, to promote public health, the Catalan government issued specific nutritional guidelines for pregnant women [20].

Currently, 92% of mothers want to breastfeed their babies but only 17% do EBF for six months [21]. Stopping BF is most often not the mother’s wish and happens earlier than desired. The most frequent causes of stopping breastfeeding are social barriers [22]. Mothers and families need support to ensure that their children receive optimal BF during the first six months of life, as well as the progressive and appropriate introduction of complementary feeding. Healthcare professionals play an essential role in providing this support [23].

The WHO recommends the support of health services in providing advice on infant and child nutrition. It is committed to supporting countries in accordance with the Global Nutrition Targets approved by Member States [24]. One of these targets is to increase the rate of EBF during the first six months of life to at least 50% [24].

In the 2019 document *Green Feeding Europe*, the Council of the European Union makes a collaborative effort with institutions to inform and involve the Green Parties of Europe, so they include the nutrition of infants and young children in their environmental programmes [12].

In the face of the climate emergency, it is crucial to understand the impact of maternal diet on health and the environment. A change towards a healthier and more environmentally friendly diet should be promoted, while increasing the rates of exclusive breastfeeding can be considered an issue related to public health, social rights, and inequality. Government investment and changes in these areas must be achieved, including longer maternity leave, spaces where women can breastfeed freely and comfortably, the monitoring of surrogate advertising, more promotion of breastfeeding and access to professionals.

To our knowledge, there are no studies on the environmental benefits of breastfeeding, or the quality and type of diet followed by postpartum women in Catalonia, Spain. Therefore, measuring the environmental benefit of breastfeeding may help protect the environment and consequently the health of the population and the planet [25, 26].

This project is split into two independent sequential projects. The results from Phase I will bolster the development of educational materials to be used in Phase II (educational intervention to improve prevalence of breastfeeding and a sustainable diet).

### Hypothesis

EBF will have smaller environmental impact than FF or mixed feeding (MF).

### General Aim

Calculate and compare the environmental impacts (climate change, water consumption and water scarcity) of three infant feeding types (EBF, FF, and MF).

#### Objectives of the study

Primary objective

- Determine the environmental impacts (climate change, water consumption and water scarcity) of EBF, MF and FF of postpartum mothers in their baby’s first month of life, taking into account the accessories used for infant feeding and mother´s diet and food consumption habits.

Secondary objectives:

- To calculate the prevalence of EBF, FF and MF after birth, and at 4–6 weeks postpartum.
- To describe the maternal diet in terms of macro, micronutrients and energy consumed.
- To compare the calories consumed by according to the type of breastfeeding, lactating mothers (EBF and MF) and non-lactating mothers (FF).
- To compare feeding types with socio-demographic and clinical data.
- To compare maternal diet and food consumption habits with feeding type and socio-demographic data.

## Methods and analysis

For this article, we used the SPIRIT reporting style for the protocol studies [27].

### Study design

This is a multicentre, cross-sectional study conducted in the Barcelona North Metropolitan Area (Catalonia, Spain) of the Catalan Health Institute (ICS), which provides universal coverage. The participating sites are the primary care settings of Sabadell, Granollers, Santa Coloma de Gramanet, Mollet, Mataró, Badalona, and Sant Adrià.

### Sample size calculation

For the calculation we took into account both the local data on prevalence of three infant feeding types at month 1 [21] and the data provided by the literature for the footprint for consumption of 1 kg powdered breastmilk substitute for the nearest geographical area. This is 11 kg CO2 equivalents and standard deviation 1 in UK/France [1]. Accepting an alpha risk of 5% and a power of 80%, in a two-tailed test, a minimum of 60 subjects are necessary in the EBF group, 60 in the mixed and in 30 in the FF group to recognize as statistically significant a difference greater than or equal to 0.7 units (One-Way ANOVA menu, StudySize 2.0) [28]. The common standard deviation is assumed to be 1 [1] (One-Way ANOVA menu, StudySize 2.0, [28]).

These 150 women (60 + 60 + 30) are the minimum number we need to recruit. However, at the time of recruitment, we do not know which breastfeeding arm each of the participating women will be in during the follow-up month. From our own previous studies [21], we know that after one month of follow-up, from an initial recruitment of 400 women, some 256 (64%) could be breastfeeding exclusively, 92 (23%) using mixed and 52 (13%) a formula feeding [21]. These expected female counts exceed the required minimums in each arm, and would allow replacement rates for losses during follow-up of up to 35%.

### Participants, inclusion and exclusion criteria

The study population consists of pregnant women (in their last month of pregnancy) from the previously mentioned primary care centres in the Barcelona North Metropolitan Area of the ICS.

The* inclusion criteria* consist of women assigned to the aforementioned centres, who are at least 16 years old, and are at one of their last pregnancy check-ups (or first postpartum visit).

The* exclusion criteria* are language barriers preventing informed consent and consent withdrawal.

### Recruitment

Midwives from the participating centres recruit women at one of their last pregnancy check-ups (or first postpartum visit) during November 2022 and May 2023. Those who meet the inclusion criteria and agree to participate in the study should be informed about the project and asked to sign the informed consent.

### Procedure

In order to calculate the environmental impacts of the different types of infant feeding, the necessary variables are first to be collected in different questionnaires. Subsequently, the impacts of the types of breastfeeding will be calculated by adding together the impacts associated with the use of accessories, artificial milk and the mothers' diet for each type of infant feeding. The questionnaires are completed by the midwife during the visit with the mother.

#### Data collection

Three questionnaires are to be prepared, using the REDCap platform [29]:

1. Recruitment visit (indicating the moment in which they are recruited, visit week 38–40): Questionnaire concerning the socio-demographical data of mothers.
2. Postpartum visit (7–10 days): Clinical data questionnaire – Newborn variables.
3. Fortieth-day visit (4–6 weeks): Questionnaire on breastfeeding accessories and mother's nutrition variables.

All study variables are described in Annex II. Recruiting midwives are to be trained on how to complete the 24-h dietary recall and to be provided with a visual food atlas as support (Annex III) to help women record ration measurements.

### Study variables

#### Dependent variables (outcomes)

*The environmental impacts* – climate change, water consumption, and water scarcity (for definitions see Annex I) – of the three infant feeding types and maternal diet:

- *CC (climate change)* will be assessed in kg CO2eq by the IPCC GWP 100 years calculations [30];
- *WC (water consumption)* will be calculated in m3 following the ReCiPE method [31], calculating direct and indirect water consumption.
- * WS (water scarcity)* will be calculated in m3eq using the AWARE method [32].

#### Independent variables

1. *Socio-demographic and clinical profile:* age; gender; relationship status; country of birth; religion; paid work; TPAL; baby’s birth weight; weeks of gestation at birth; maternity leave; partner leave to care for the baby; maternal pathology and neonatal pathology.
2. *Newborn variables:* type of feeding in hospital and at 1 month of the child’s life; in the case of MF or FF, indicate how much infant formula you feed your baby daily, in ml; number of bottles or syringes of infant formula you give your baby daily; how many ml does each bottle or syringe have.
3. *Breastfeeding accessories:* use (and quantity in case of several) of a breast pump; milk storage containers; bags to store milk; nipple shields; a nursing supplementer; cans of infant formula; bottles; brushes to clean the bottles; a bottle warmer; a bottle sterilizer; paediatric nasogastric tubes; reusable nursing pads and disposable nursing pads.
4. *Mother's nutrition variables: a) Eating habits:* type of diet; taking any vitamin supplements; usual place to purchase food, transport of food and distance from home; type of energy used for cooking; members of the family who usually cook and how much of the food is thrown away? *b) Recording of the mother's daily diet (recall 24 h)* [33, 34] (validated in other studies [35, 36]), *and food consumption habits:* type of food: portions, packaging, type of cooking, characteristics of the product (fresh, frozen), origin of the product, condiments and beverages.

The maternal diet will be measured in terms of macronutrients (carbohydrates, proteins and fatty acids in grams), micronutrients (calcium, magnesium and in milligrams, vitamin D in micrograms) and energy consumed in kcal).

## Data analysis plan and expected outcomes

### Data analysis plan

The environmental impacts of the mother's daily diet and feeding type, focusing on CO_2_ emissions (climate change) and water consumption (as well as water scarcity), will be calculated based on ISO 14040 and 14,044, and other related ISOs [30–32]. To analyse the environmental data, a comparative life cycle study will be conducted considering the carbon footprint and, additionally, resource depletion (water consumption and scarcity) as environmental indicators, among others [30–32].

The environmental impacts (climate change, water consumption, and water scarcity) of the infant feeding types and maternal diet will be calculated using the IPCC [30], ReCiPE [31], and AWARE [32] validated methods, respectively.

**Fig. 1:**
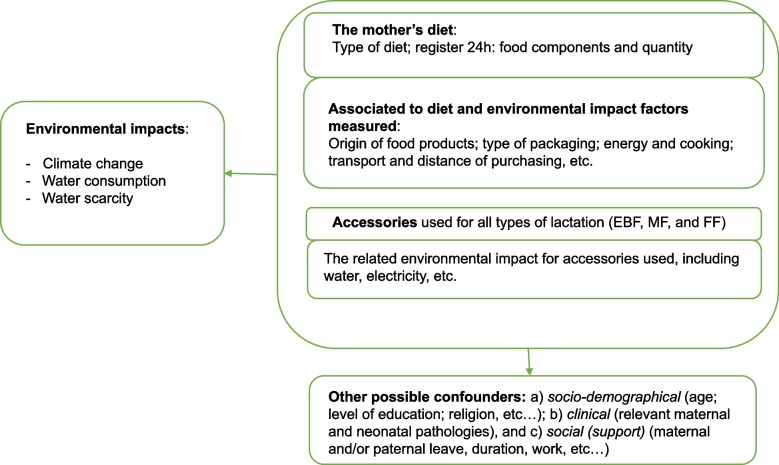
Environmental impact factors considered in the GREEN MOTHER I project. Legend: EBF – exclusive breastfeeding; MF – mixed feeding; FF – formula feeding

The results of environmental impacts will be presented considering the following groups (Fig. 1):

- Feeding type and accessories used for infant feeding: EBF, FF, or MF
- The mother's diet and food consumption habits.

For the descriptive statistics, the qualitative variables will be described with their absolute and relative frequencies and quantitative variables with their mean and standard deviation or with their median and quartiles. In the comparison of proportions, we will use the Pearson Chi Square test and in the comparisons in quantitative variables the analysis of variance or the Kruscal-Wallis test (with post-hoc analyses), depending on the conditions of application. The level of significance will be 5% and the analyses will be performed with the statistical package STATA v.18.

### Expected outcomes

1. The environmental impacts (climate change, water consumption and water scarcity) are higher in FF compared to EBF and MF.
2. The environmental impacts (climate change, water consumption and water scarcity) due to an increase in maternal diet to compensate for breastfeeding are insignificant.

### Ethics and safety

The study follows the guidelines of the Declaration of Helsinki regarding the bioethical principles of clinical research. The research protocol has been approved by the Research Ethics Committee (CEI) of the Jordi Gol Institute of Primary Care Research (IDIAP), as well as those of the reference hospitals participating in the study: Hospital Parc Taulí, Hospital General de Granollers, Hospital Germans Trias i Pujol and Hospital de Mataró.

The data collected by questionnaires on REDCap are to be encrypted. The variables needed to conduct the study have been obtained directly from the project participants through their consent, in accordance with the provisions of Articles 6.1.a) and 9.2.a) of the GDPR.

The REDCap platform is used to carry out the project. It is hosted on the institution's servers and its security measures are determined by the institution. The data is stored on the local web server where the institution has installed the software and is therefore only accessible on computers that have a trusted connection to it via VPN (Virtual Private Network) and secure credentials (certificates, RSA keys or complex passwords). A system has been incorporated so that only the application service can send data to the back office through a firewall that only allows requests from the application's IP addresses. The web server has the X-Frame-Options HTTP header setting enabled with the value "same-origin" to prevent clickjacking attacks.

Compliance with the Patient Autonomy Law is ensured and the data are collected anonymously in compliance with EU regulation 2016/679. The ICS is the data controller and the current study data owner.

## Discussion

The main aims of this study (Phase I) are to explore: 1) environmental impacts of three types of infant feeding including accessories used and maternal diet; 2) characteristics of maternal diet with food consumption habits and 3) socio-demographic and clinical factors associated to three infant feeding types. As per the results of this study, a report will be drawn up with environmental data on the mothers' diet and the accessories used during infant feeding. This will also serve as the basis of the documentation for the following GREEN MOTHER Phase II to evaluate the effectiveness of a multimodal educational and support intervention on EBF prevalence, diet and the environmental impact of different types of infant feeding.

For this intervention, we create the following resources:

- A Guide on best practices in breastfeeding, nutrition and sustainability based on scientific evidence that will serve to unify criteria and update knowledge for midwives and other health workers.
- Other informative tools (posters, leaflets, video tutorials) for excellence and best practices in breastfeeding care and sustainable nutrition will be prepared and disseminated among professional communities to create environmental awareness both in health workers and the general public at the national level.

### Strengths of the Study

To the best of our knowledge, this research represents the first endeavour of its nature in Spain, boasting a considerable sample size of 429 participants. It furnishes authentic insights into the behaviours of postpartum mothers throughout the initial month of their infants' lives, scrutinized through the lens of sustainability. This investigation has potential to guide mothers in their choices concerning infant feeding, acknowledging the smaller environmental footprint associated with breastfeeding.

### Study Limitations

The usual recommendation for 24-h dietary recall at the individual level is three days. In our case, as data is analysed in an aggregate manner, without carrying out intrapersonal analysis, recording one day seems the best option for adapting the research which intends to collect the minimum amount of data necessary to fulfil the objectives. Moreover, we have high risk of losing participants, attributable to the peculiarities of the vital period under study and the oversaturated care demands in the public health System.

### Dissemination

The results will be disseminated in the professional community and general public through continuing education courses, talks, colloquiums in the community, and presentations at conferences and congresses.

We plan to prepare three scientific articles:

- 1 article on Protocol (Phase I), the current one
- 2 articles on the results: 1) on the infant feeding type prevalence and maternal diet at 1 month of postpartum in journals in the field of nutrition and health and 2) the environmental impact of infant feeding to be sent to multidisciplinary journals on infant feeding, nutrition, health and environmental protection.

Moreover, we plan to present our projects and results in scientific conferences for health professionals in the region, at local and state level, annual meetings of gynaecologists and midwives, in the annual congress of midwives and congresses on primary care.

### Supplementary Information

Supplementary material.

## Data Availability

Not applicable.
