# Supplementary material for: Environmental impact of infant feeding type, accessories used and maternal dietary habits: The GREEN MOTHER-I project, a cross-sectional study protocol
Source: Nutr J. 2024 Aug 21;23:97. doi: 10.1186/s12937-024-01000-9 (PMC11337649; doi:10.1186/s12937-024-01000-9)
Supplement: Supplementary file 1 — Supplementary material. [file 12937_2024_1000_MOESM1_ESM.docx]

**ANNEX I: Definitions of infant feeding and environmental impacts types used in the study:**

***Infant feeding types:***

1. **Excusive breastfeeding (EBF)^[[1]](#footnote-1)^** means feeding your baby only breast milk, not any other foods or liquids (including infant formula or water), except for medications or vitamin and mineral supplements.
2. **Formula feeding (FF)^[[2]](#footnote-2)^** includes any infant formula or any animal milk (cow milk, goat milk, evaporated milk and reconstituted powdered milk.
3. **Mixed feeding (MF)^2^ –** includes human breast milk, other than any formula or any liquid animal milk.

***Environmental impacts:***

**1) Climate change (CC)** refers to the global warming potential caused by increased greenhouse gas concentrations, which increases the global temperature and the frequency and intensity of climatic events.

**Carbon footprint (CF)** is a measure of the amount of carbon dioxide released into the atmosphere as a result of the activities of a particular individual, organization, or community.

***Water footprint (WF)*** is an environmental indicator that measures the volume of total water used to produce and serve consumption by an individual, community, product or company. It shows the human impact on global water resources and provides a base of knowledge on which strategies should be created to minimize negative impacts on these resources^[[3]](#footnote-3)^.

The WF calculation comprises various categories: blue WF (surface and groundwater volume), green WF (rainwater volume), and grey WF (polluted freshwater volume) utilized in product production^[[4]](#footnote-4)^.

2) ***Water consumption (WC)*** refers to the increase in water consumed. It includes direct water consumption (the consumption of water in the product system) and indirect water consumption (the consumption of water in the value chain of the input used in the production system)^3^.

3) ***Water scarcity (WS)*** refers to the shortage of water, an imbalance between the supply and demand of water, and also good quality of water. Causes of water scarcity include overuse of water, loss of water due to leakages, excessive use of water for washing purposes, taps left open, etc.

**ANNEX II: Study variables and categories of replies**

These variables were obtained through the survey designed and approved by the institutional ethical committee (see Methods part) (REDCap).

1. ***Socio-demographic and clinical profile:***

• Age

• Gender: female, male, non-binary, other

• Relationship status: male partner, female partner, non-binary partner, no partner, other

• Country of birth:

• Religion: Islam, Hindu, Buddhist, Christian, Jewish, others.

• Paid work: yes/no

• TPAL: births, preterm births, abortions, living children

• Baby’s birth weight: in grams

• Weeks of gestation at birth: weeks

• Maternity leave: yes/no, expected duration in weeks

• Partner leave to care for the baby: yes/no, expected duration in weeks

• Maternal pathology: insulin-induced diabetes, type II diabetes, hypertension, digestive disease, immunological disease, endocrine disease, mental illness, breast surgery, others

• Neonatal pathology: low birth weight baby, premature, jaundice/high bilirubin, newborn hospitalization

1. ***Newborn variables:***

• Type of feeding in hospital: EBF, MF, FF.

• Type of feeding at 1 month of the child’s life: EBF, MF, FF.

• In the case of MF or FF, indicate how much infant formula you feed your baby daily, in ml:

• Number of bottles or syringes of infant formula you give your baby daily: from 1 to more than 8 a day.

• How many ml does each bottle or syringe contain: 10–150 ml

1. ***Breastfeeding accessories:***

• Do you use a breast pump: yes/no

• Do you use milk storage containers: yes/no

• Total number of milk storage containers you own:

• Do you use bags to store milk: yes/no

• How many bags do you use each week:

• Do you use nipple shields: yes/no

• Do you use a nursing supplementer: yes/no

• How many cans of infant formula do you use weekly: zero or number per week (based on a 800 gr can)

• Do you have bottles: yes/no and number

• Do you use brushes to clean the bottles: yes/no

• Do you use a bottle warmer: yes/no

• Do you use a bottle sterilizer: yes/no

• Do you use paediatric nasogastric tubes: yes/no

• Reusable nursing pads: yes/no

• Disposable nursing pads: yes/no and how many disposable nursing pads do you use per week.

1. ***Mother's nutrition variables:***

*a. Eating habits*

• What type of diet do you follow: no special diet, vegetarian, vegan, gluten-free, food allergies, therapeutic diet (diabetes, high blood pressure, dyslipidaemia, etc.)

• Do you take any vitamin supplements: yes/no

• Where do you usually buy food: supermarket, take away, small business (neighbourhood, market), local product (km 0) usually, local product (km 0) less than 50%, local product (km 0) more than 50%

• How do you usually go shopping: car 0–5 km, car 6–10 km, car > 11 km, walking, public transport

• Type of energy in your kitchen: butane gas, natural gas, electricity, biomass (wood)

• Members of the family who usually cook: mother, partner, both, others

• How much of the food do you end up throwing away: from < 5%, 6–10%, 11–24%, > 25%

*b. Record of the mother's daily diet (recall 24 hours)*

• Diet. “24-hour food record. 24hR”: The three main meals and snacks (before breakfast, mid-morning, afternoon, and before bed)

• Type of food: portions, packaging, type of cooking, characteristics of the product (fresh, frozen), origin of the product, condiments and drink

• Packaging: glass, tetra brick, plastic, paper, no packaging

• Characteristics of the food: fresh, frozen, pre-cooked, canned, preserved in glass

• Cooking methods: raw, boiled, steamed, fried, baked, or grilled

• Sauces and condiments: mayonnaise, ketchup, tomato, mustard, soy sauce, oil, vinegar, butter

• Beverages: water, carbonated soft drinks (Coca-Cola, lemonade, etc.), packaged juices, alcoholic beverages, vegetable milk or animal milk

• Food origin: local, Catalonia, Europe/Mediterranean, unknown, other

• Food measurement: small (100 gr), medium (200 gr), large (300 gr); fruit: ½ piece small portion, 1 piece (medium portion), 2 large pieces (see the Food Atlas in Annex III).

**ANNEX III: The Nutrition Atlas**

This Nutrition Atlas was developed by the GREEN MOTHER project research team and will be used in the study:

VEGETABLES (example in English):


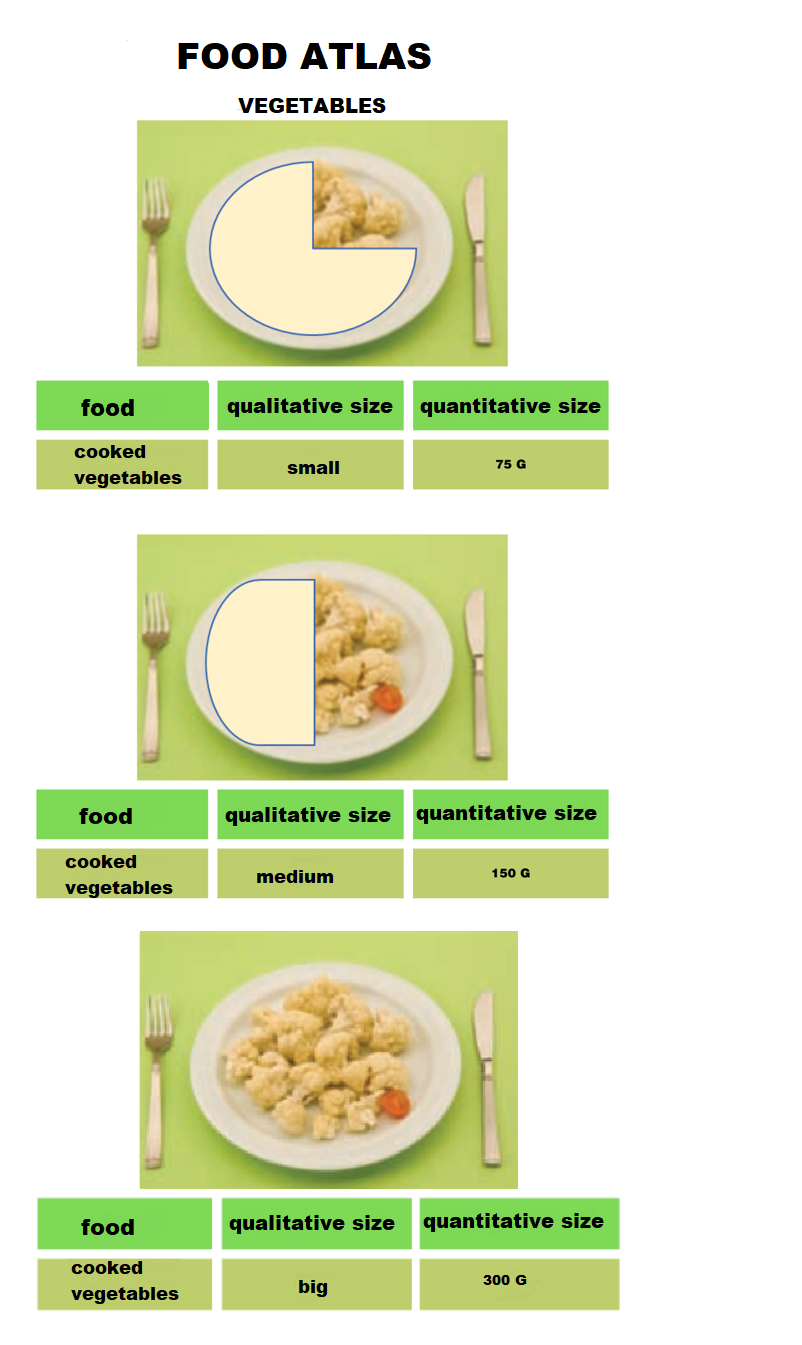


*Note:* The Food Atlas guidelines for portions were adapted from: <http://www.aprendizdediabetes.es/guia-roche-2018> and <https://www.aesan.gob.es/AECOSAN/docs/documentos/seguridad_alimentaria/gestion_riesgos/atlas_enalia.pdf>

1. https://www.cdc.gov/nutrition/infantandtoddlernutrition/definitions.html [↑](#footnote-ref-1)
2. 2 Indicators for assessing infant and young child feeding practices: definitions and measurement methods. Geneva: World Health Organization and the United Nations Children’s Fund (UNICEF), 2021. https://creativecommons.org/licenses/by-nc-sa/3.0/igo. [↑](#footnote-ref-2)
3. Hoekstra AY, Mekonnen MM. The water footprint of humanity. Proc Natl Acad Sci U S A. 2012;109:3232–7. [↑](#footnote-ref-3)
4. Mekonnen MM, Hoekstra AY. A Global Assessment of the Water Footprint of Farm Animal Products. Ecosystems. 2012;15:401–15 [↑](#footnote-ref-4)
